# Experience-dependent modulation of alpha and beta during action observation and motor imagery

**DOI:** 10.1186/s12868-017-0349-0

**Published:** 2017-03-06

**Authors:** Paula M. Di Nota, Julie M. Chartrand, Gabriella R. Levkov, Rodrigo Montefusco-Siegmund, Joseph F. X. DeSouza

**Affiliations:** 1 Department of Psychology, York University, Toronto, ON M3J 1P3 Canada; 2 Neuroscience Graduate Diploma Program, York University, Toronto, ON M3J 1P3 Canada; 3 Centre for Vision Research, York University, 4700 Keele Street, Toronto, ON M3J 1P3 Canada; 4 Department of Biology, York University, Toronto, ON M3J 1P3 Canada; 5 Interdisciplinary Studies, York University, Toronto, ON M3J 1P3 Canada

**Keywords:** EEG, Motor imagery, Action observation, Dance, Plasticity, Alpha, Beta, Sensorimotor, Expertise, Familiarity

## Abstract

**Background:**

EEG studies investigating the neural networks that facilitate action observation (AO) and kinaesthetic motor imagery (KMI) have shown reduced, or desynchronized, power in the alpha (8–12 Hz) and beta (13–30 Hz) frequency bands relative to rest, reflecting efficient activation of task-relevant areas. Functional modulation of these networks through expertise in dance has been established using fMRI, with greater activation among experts during AO. While there is evidence for experience-dependent plasticity of alpha power during AO of dance, the influence of familiarity on beta power during AO, and alpha and beta activity during KMI, remain unclear. The purpose of the present study was to measure the impact of familiarity on confidence ratings and EEG activity during (1) AO of a brief ballet sequence, (2) KMI of this same sequence, and (3) KMI of non-dance movements among ballet dancers, dancers from other genres, and non-dancers.

**Results:**

Ballet dancers highly familiar with the genre of the experimental stimulus demonstrated higher individual alpha peak frequency (iAPF), greater alpha desynchronization, and greater task-related beta power during AO, as well as faster iAPF during KMI of non-dance movements. While no between-group differences in alpha or beta power were observed during KMI of dance or non-dance movements, all participants showed significant desynchronization relative to baseline, and further desynchronization during dance KMI relative to non-dance KMI indicative of greater cognitive load.

**Conclusions:**

These findings confirm and extend evidence for experience-dependent plasticity of alpha and beta activity during AO of dance and KMI. We also provide novel evidence for modulation of iAPF that is faster when tuned to the specific motor repertoire of the observer. By considering the multiple functional roles of these frequency bands during the same task (AO), we have disentangled the compounded contribution of familiarity and expertise to alpha desynchronization for mediating task engagement among familiar ballet dancers and reflecting task difficulty among unfamiliar non-dance subjects, respectively. That KMI of a complex dance sequence relative to everyday, non-dance movements recruits greater cognitive resources suggests it may be a more powerful tool in driving neural plasticity of action networks, especially among the elderly and those with movement disorders.

## Background

The three facets of movement processing—action observation (AO), kinaesthetic motor imagery (KMI), and execution—have been shown to recruit a common ‘mirror neuron’ network of brain regions in both humans [1, 2] and non-human primates [3]. Each of these processes is vital in facilitating motor learning in general and especially for dance movements, which require transformation of multisensory inputs into highly specific and complex motor outputs that are reproduced with high fidelity (for review, see [4, 5]). As a result of this experience, activation of the mirror network and associated brain regions is modulated by dance experience [6, 7] to facilitate subsequent motor learning [8].

These and other evaluations of motor representations for complex movements that cannot physically be performed during neuroimaging have utilized tasks involving AO and/or KMI from an internal first-person perspective. During four functional magnetic resonance imaging (fMRI) scans conducted over a 34-week period, recent investigations conducted by our lab have employed both AO and KMI tasks for a novel piece of choreography that was rehearsed and performed by professional ballet dancers. Consistent with findings from Cross et al. [9], we found plasticity in the motor representation for this specific dance sequence coded in sensorimotor brain regions over time, as well as decreased activation in the extrastriate body area during movement of the foot in dancers relative to novice controls [10, 11].

While most of the evidence for experience-dependent plasticity has come from fMRI studies, valuable insight into the temporal dynamics of sensorimotor processing has been revealed with electroencephalography (EEG). With respect to action processing, the rolandic ‘mu’ alpha rhythm (9–13 Hz) recorded directly from midline primary motor and somatosensory cortices has been shown to decrease in power relative to rest, or desynchronize, when planning, imagining and executing movements [12], with concurrent desynchronization of the beta (13–30 Hz) frequency band observed in motor cortex [13]. Alpha desynchronization is also observed in other brain areas under conditions of increased attentional and cognitive effort, putatively reflecting suppression of task-irrelevant areas to enhance performance (for review, see [14] and [15]). Within the alpha band there is typically a maximal amplitude frequency known as the individual alpha peak frequency (iAPF) that is typically lower (or slower) in the elderly [16] and higher (or faster) during states of cognitive preparedness [17] and under increased cognitive demand [18].

Investigations on the influence of dance expertise on EEG activity are sparse, as most expert studies assess either proficiency in task performance, various types of athletes (for review, see [19]) or the influence of aerobic exercise on EEG activity among non-experts [20]. However, a study by Ermutlu and colleagues [21] compared resting EEG activity across delta (0.5–4 Hz), theta (4–8 Hz), alpha, and beta frequency bands in fast ball sport athletes and dancers. They correctly predicted that athletes, who have to quickly anticipate observed movements and adapt their own motor responses, would show higher power in slower bands, and dancers experienced at practicing rhythmic, imaginative and repetitive movements would show higher power in the alpha and beta bands. With respect to task-related activity during movement processing, Orgs et al. [22] recorded EEG from expert dancers and non-experts while they observed dance and non-dance movements. They found no group differences during the latter condition, and alpha desynchronization among dancers only when observing dance movements. It remains to be seen whether similar experience-dependent modulation of the alpha or beta bands occur during KMI of dance or non-dance movements, and is one of the primary goals of the present investigation.

Expanding upon the existing literature, the current study sought to compare differences in alpha and beta power during AO and KMI of a ballet dance sequence among expert dancers that were both familiar and unfamiliar with the genre, as well as non-dancers, in order to disentangle the effects of familiarity and expertise more generally on action processing of dance movements. Similar to Orgs et al. [22], we also had participants perform KMI for non-dance movements in order to examine possible transference of expertise effects.

## Methods

### Participants

Subjects were recruited through the York University Undergraduate Research Participant Pool (URPP) and compensated with course credit. The research study was approved by the Office of Research Ethics’ Human Participants Review Committee (Certificate # 2013-211) and in accordance with the Declaration of Helsinki. Eligibility requirements included self-reported right-handedness and no uncorrected visual or neurological problems.

The advertised study called for participants with at least 2–5 years of experience in any genre of dance or related craft including gymnastics, figure skating, or sports. A total of 92 participants were tested, but 31 were excluded for the following reasons: lack of event markers in EEG data (n = 9), demographic information not provided (n = 9), reported neurological problems (n = 1), reported left-handedness (n = 4), poor EEG data quality (n = 6), and no registered alpha peak values (n = 2). A total of 61 participants (48 female) between the ages of 18 and 37 (*M* = 20.7, *SD* = 4.22) were analyzed. Participants were divided into three groups based on their self-reported experience: ballet dancers (n = 25, 22 females, mean age = 20.56, *SD* = 4.0, mean years of experience = 10.44, *SD* = 4.3), non-ballet dancers with experience in other genres of dance (n = 21, 15 females, mean age = 20.67, *SD* = 3.6, mean years of experience = 5.57, *SD* = 3.1), and non-dancers (n = 15, 11 females, mean age = 21.07, *SD* = 5.5, mean years of experience = 5.8, *SD* = 4.6).^1^ Participants in both dance groups were required to have at least 2 years of experience. Non-ballet dance genres included belly dancing, hip hop, ballroom, Salsa, tap, modern, lyrical, acro, cultural, folk, Bollywood, jazz, break dancing, and contemporary. Non-dance subjects were not required to be experts in any particular craft, but included subjects with at least 2 years of experience in skills such as figure skating (n = 5), gymnastics (n = 4), martial arts (n = 1) and music (n = 1), as well as non-experts (n = 4).

### Procedure

After providing their informed consent to participate in the study, participants were measured and fitted with the EEG headset according to the International 10–20 System of electrode placement. Participants were instructed to remain as still as possible during the experimental tasks and were provided with ear bud headphones to hear auditory stimuli. The computer-based experiment began with a brief demographic survey and baseline recordings followed by three tasks: (1) action observation (AO) and (2) kinaesthetic motor imagery (KMI) of a ballet dance sequence, and (3) the KMI portion of the Visual and Motor Imagery Questionnaire (VMIQ-2) [23]. In order to minimize differences in dance imagery across participants, the AO task was performed first and allowed subjects to view the dance sequence as many times as needed in order to form a clear mental image for the subsequent KMI task.

#### Baseline

Four 15-s resting conditions were performed in random order to facilitate baseline correction of subsequent tasks: eyes open without music (BEO), eyes open with music (BEOM), eyes closed without music (BEC), and eyes closed with music (BECM). Participants were instructed to clear their minds and to follow instructions as they were given. A picture of a closed eye appeared on screen during the eyes closed conditions to remind subjects to keep their eyes closed in the event they opened them during the task (vertical visual angle = 13.6°), and a central fixation cross (visual angle = 0.98°) appeared during the eyes open conditions. Audio prompts cued subjects to open and close their eyes at the beginning and end of each baseline condition where appropriate. The music conditions (BECM, BEOM) played the first 15 s of Bach’s *Goldberg Variations 988, Variation 1* to match the cadence and genre of the music in the AO and KMI tasks.

#### AO task

Following the baseline recordings, participants repeatedly viewed an 8-s video clip of a choreographed ballet dance. The clip was taken from a 6.92-min video that was filmed during a practice at the Walter Carsen Centre for The National Ballet of Canada from Bar and DeSouza’s [10] study, and was played full screen on the computer monitor (vertical visual angle = 13.6°). The video clip featured one female dancer in the foreground and the music was from Bach’s *Concerto in C Major*. Participants were asked to closely observe the choreography performed by the female dancer in order to be able to imagine themselves performing it as accurately and precisely as possible during the next experimental task. Because participants were fit for EEG recording during the experimental session, motor performance was not behaviourally assessed in the laboratory. Instead, participants were asked to rate how confident they were that they could perform the dance with complete accuracy and precision if required to on the following scale modelled after the VMIQ-2 scale [23]: 1 = *Perfectly accurate and in time with the music*, 2 = *Clear and reasonably accurate and in time with the music*, 3 = *Moderately accurate and in time with the music*, 4 = *Vaguely accurate and in time with the music and dim*, 5 = *No accuracy at all and unable to keep time with the music*. The clip was initially shown 10 times (Block 1) with a 1-s interstimulus interval and fixation cross (visual angle = 0.98°) followed by a prompt for a confidence rating. A participant response of 1 or 2 (i.e., indicating high confidence) would induce the end of the AO task, and participants were asked if they would like to view the clip a final five times (Block 2). A response of 3–5 would automatically present an additional block that played the dance clip five times (Block 2). Following this shorter block, participants were once again asked to rate their confidence that they could physically perform the dance if required to. If participants respond below threshold (i.e., 3–5), they could watch the shorter block for a maximum of four times before automatically proceeding to the next experimental task. Participants could view the clip a maximum of 30 times across 5 blocks. The AO portion of the experiment lasted approximately 5–15 min.

#### KMI task

Following the AO task, participants were asked to visualize themselves performing the dance sequence they had just learned with their eyes open and closed. Four blocks (2× eyes open and 2× eyes closed) of 25 trials each were randomly presented, resulting in a total of 100 KMI trials. During each trial, participants heard the music that had accompanied the dance clip during the AO task. Participants were asked to imagine themselves performing the dance in time with the music from an internal, first-person perspective and to feel themselves executing the movements as opposed to visualizing from a third-person perspective (i.e., ‘seeing’ themselves perform the dance from an external perspective). The difference in visualization perspectives was explained during initial briefing as well as in the written instructions presented on screen to ensure the proper engagement in motor, and not visual, imagery [24]. The dance clip was played once before each block to refresh the participant’s memory and ensure that they were accurately imagining the dance. Similar to the baseline procedure, a picture of a closed eye appeared on screen during the eyes closed condition and a central fixation cross was provided during the eyes open condition. Participants were provided with audio prompts cuing them to open and close their eyes when appropriate. As with the AO task, participants were asked to rate how clearly and vividly they were able to feel themselves performing the dance sequence at the end of each block. Ratings were once again given on a 5-point scale as follows: 1 = *Perfectly clear and as vivid* (*as normal vision or feel of movement*), 2 = *Clear and reasonably vivid*, 3 = *Moderately clear and vivid*, 4 = *Vague and dim*, 5 = *No image at all, you only “know” that you are thinking of the skill*. Once an answer was recorded, participants were given the opportunity to take a break before beginning the next block. Each block lasted 3.5–4 min and the KMI task lasted approximately 15–20 min.

For the purpose of the present study only eyes closed KMI conditions will be included in the analysis so as to compare them to the next task, which was only performed with eyes closed.

#### VMIQ-2 task

In order to compare each participant’s EEG activity and subjective perceptual level of vividness and clarity to KMI for everyday, non-dance movements, the final experimental task required completing the third portion (KMI from a first-person perspective) of the VMIQ-2 [23]. Participants were instructed to perform KMI of 12 everyday, non-dance movements (Table 1) while eyes were closed in accordance with VMIQ-2 instructions. For each item, the requested action was written on the screen and the participant was instructed to first close their eyes and then press the spacebar when they were ready to begin visualizing. Participants were instructed to spend as much time as they needed to form a clear and accurate image of feeling themselves complete each action, and to press the spacebar once they had finished visualizing. Upon this second button press, participants were asked to rate how clear and vivid their KMI was for that item using the same 5-point scale from previous tasks (i.e., 1 = *Perfectly clear and vivid*, 5 = *No image at all*). As this portion of the experiment was self-paced, completion time varied from 1 to 3 min (average duration = 4.57 min, *SD* = 4.25 min). Valid iAPF values were obtained from 59 subjects^2^ who were included in the final analyses.

Following completion of the VMIQ-2, participants were asked if they felt that KMI was easier to complete with eyes closed or eyes open and their responses were recorded. Finally, the EEG headset was removed and participants were debriefed and thanked for their participation. The entire experiment from entrance to exit lasted approximately 40–55 min.

**Table 1:** Visualized movements included in the Vividness of Movement Imagery Questionnaire (VMIQ-2)

Item
1. Walking
2. Running
3. Kicking a stone
4. Bending to pick up a coin
5. Running up stairs
6. Jumping sideways
7. Throwing a stone into water
8. Kicking a ball in the air
9. Running downhill
10. Riding a bike
11. Swinging on a rope
12. Jumping off a high wall

The complete VMIQ-2 [23] requires participants to imagine each of these items in turn under three conditions: internal visual imagery, external visual imagery, and kinesthetic (i.e., motor) imagery. The present study was only interested in evaluating kinesthetic motor imagery, so participants were asked to imagine themselves performing each of the items listed with their eyes closed and as though they could “feel [them]self doing the movement” as per the VMIQ-2 instructions [23]. Once they had formed a clear motor image of each item, participants were asked to rate how clearly and vividly they could feel themselves performing the movement on the following scale: 1 = *Perfectly clear and as vivid (as normal vision or feel of movement)*, 2 = *Clear and reasonably vivid*, 3 = *Moderately clear and vivid*, 4 = *Vague and dim*, 5 = *No image at all, you only “know” that you are thinking of the skill*

### Data acquisition, processing and analysis

EEG data were collected using a wireless 14-channel (AF3, AF4, F3, F4, F7, F8, FC5, FC6, T7, T8, P7, P8, O1, O2) Emotiv EPOC EEG Neuroheadset and recorded with accompanying TestBench software (Emotiv Systems, 2012, San Fransisco, CA). The headset has two reference locations (at M1 and M2), a sampling rate of 128 Hz with 16-bit ADC resolution, and 0.02–45 Hz resolution with digital 5th-order sinc notch filters at 50–60 Hz. The Emotiv neuroheadset has been validated [25] and provides several ecological advantages to traditional research-grade EEG systems. These include affordability and portability to record EEG in environments outside of the laboratory, including a dance studio where neural activity can be evaluated on-site [26]. All experimental tasks were presented on a 23ʺ flat screen monitor (Dell P2312H) and configured and presented by MediaLab (v2012.4.119, Blair Jarvis for Empirisoft Co., New York, NY). Data markers were sent from MediaLab to TestBench via a Virtual Serial Port Driver (Version 7.1, Eltima Software, 2013, Bellevue, WA). Preprocessing of the EEG data was conducted with the Fieldtrip toolbox (Version 20131117, [27]) and Matlab (Version 7.10.0.499, The MathWorks, Inc., Natick, MA), and statistical analyses were performed with SPSS (Version 22, IBM Co., Armonk, NY).

For each participant, raw EEG data was first segmented according to data markers coded for events of interest (i.e., experimental tasks), and these segments were further divided into 2-s epochs prior to being bandpass filtered (1–50 Hz), demeaned, and detrended. Next, artifacts were rejected by visual inspection (*M* = 51 trials, *SD* = 18.5 out of an average 483.3 trials, *SD* = 28.5) and independent component analyses were performed to eliminate contamination of the EEG signal from eye movements and blinks (*M* = 2.15, *SD* = 0.60 components eliminated out of 14 total components). Data were fast Fourier transformed and iAPF (Hz), alpha peak power (μV^2^), and average beta power across the 13–30 Hz range were computed for each participant, experimental task and electrode.

To normalize the variances of power data among electrodes, alpha and beta power values were log transformed and baseline corrected with the appropriate baseline condition (i.e., AO task baseline corrected with BEOM; KMI and VMIQ-2 tasks baseline corrected with BECM) prior to statistical analyses. The following equations were used to compute log task-related alpha peak power (herein referred to as “alpha”) and log task-related beta power (herein referred to as “beta”) modeled from Gonzalez-Rosa et al. [28]:

$${\text{alpha}}\;{\text{power}} = { \log }\;{\text{alpha}}\;{\text{peak}}\;{\text{power}}\;\left( {\text{task}} \right)-{ \log }\;{\text{alpha}}\;{\text{peak}}\;{\text{power}}\;\left( {\text{baseline}} \right)$$

$${\text{beta}}\;{\text{power}} = { \log }\;{\text{beta}}\;{\text{power}}\;\left( {\text{task}} \right)-{ \log }\;{\text{beta}}\;{\text{power}}\;\left( {\text{baseline}} \right)$$

Alpha power was not subdivided into the rolandic mu (9–13 Hz) frequency, as it is typically recorded from central (C3, C4, Cz, Fz) electrode sites that are not included in the Emotiv neuroheadset used in the current study. Instead, we isolated the iAPF within the traditional 8–12 Hz bandwidth and focused statistical analyses on electrode sites overlying available and relevant sensorimotor areas including bilateral frontocentral (FC5, FC6) motor association cortex [29], superior temporal cortex (T7, T8) involved in perceptual learning [30] and sensory-guided KMI [31], inferior posterior temporal gyrus (P7, P8), and primary visual occipital cortex (O1, O2) [32].

All statistical analyses including the factor of Electrode [FC5, T7, P7, O1, O2, T8, P8, FC6] employed a repeated measures analyses of variance (RM-ANOVA) design. For analyses of the AO task evaluating the factor of Time [Blocks 1–5], a mixed model structure was applied to the RM-ANOVA to account for the different number of blocks performed between subjects, which would not be adequately corrected for in a RM-ANOVA. Sphericity was assessed for each within-subject factor with Mauchly’s Test and violations were adjusted by reporting degrees of freedom and significance values with Huynh-Feldt corrections (*p*_*HF*_) [33]. Least-significant difference (LSD) significance values are reported (*p*) for main and interaction effects where sphericity is assumed, for pairwise comparisons of 2-level factors, and for one-way between-group ANOVAs. Pairwise comparisons of main effects with more than 2 levels (i.e., Group, Electrode or Time) and simple effects of significant interactions report Bonferroni corrections for multiple comparisons (*p*_*Bonf*_). Effect sizes (partial eta squared, η^2^) are reported where appropriate. Exploratory Pearson correlations were performed on data (collapsed across all factors) as secondary analyses reported in-text with LSD *p* values. The results of each task will be presented in turn in the following order: analyses of demographic data and confidence ratings, iAPF, alpha power and beta power.

## Results

### Baseline analyses

**Fig. 1:**
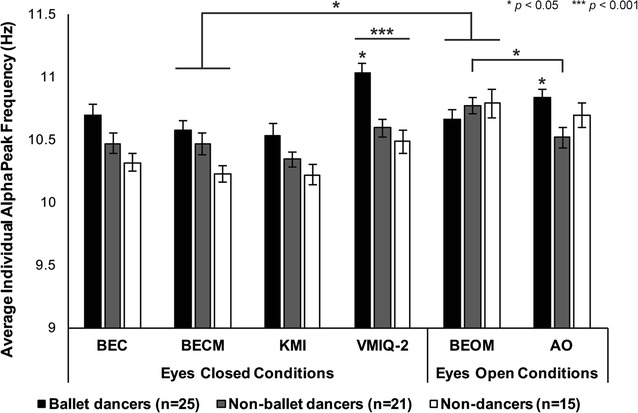
Individual alpha peak frequency (iAPF) during action observation and motor imagery. The highly familiar ballet dance group showed faster iAPF during the AO task relative to both non-ballet dance (*p* = 0.029) and non-dance (*p* = 0.018) groups, and also demonstrated faster iAPF than non-ballet (*p* = 0.049) and non-dance groups (*p* = 0.027) during the VMIQ-2 task. No significance between group effects were observed during the KMI task, and all participants had significantly faster iAPF during the VMIQ-2 task relative to the KMI task (*p* = 0.000). *Error bars* represent SEM

To evaluate possible between-group differences in baseline iAPF, log alpha peak power, and/or log beta power, three separate RM-ANOVA’s were performed with the factors of Condition (4) × Electrode (8) × Group (3). For iAPF, a main effect of Condition [*F*(3, 174) = 3.616, *p* = 0.014, η^2^ = 0.06] revealed faster iAPF during BEOM relative to BECM only (*p*_*Bonf*_ = 0.024) (Fig. 1). A main effect of Electrode just met significance criteria [*F*(7, 406) = 2.033, *p* = 0.050, η^2^ = 0.03] with no significant Bonferroni-corrected pairwise comparisons. As expected, alpha power was higher during both eyes closed conditions (BEC, BECM) relative to eyes open (BEO, BEOM) in all electrodes [Condition × Electrode: *F*(12.1, 700.9) = 49.085, *p*_*HF*_ = 0.000, η^2^ = 0.46], as was beta power in parietal (P7: *p*_*Bonf*_ = 0.002, P8: *p*_*Bonf*_ = 0.000) and occipital (O1 and O2: *p*_*Bonf*_ = 0.000) electrodes [Condition × Electrode: *F* (13.0, 25.0) = 31.608, *p*_*HF*_ = 0.000, η^2^ = 0.35]. No significant between-group differences were observed for iAPF, alpha or beta power (*p* > 0.05).

Task-related changes to alpha and beta power reported below subtract baseline in their calculations, so we will report task-related changes to iAPF relative to baseline here. To evaluate matched conditions with eyes open and music, we compared BEOM and AO iAPF with a 2 (Task) × 8 (Electrode) × 3 (Group) RM-ANOVA, which revealed slower iAPF during AO in non-ballet dancers only [*p*_*Bonf*_ = 0.016, *F*(2, 58) = 3.093, *p* = 0.053, η^2^ = 0.03]. To evaluate task-related changes to iAPF during eyes closed tasks and conditions, we compared BEC, BECM, KMI and VMIQ-2 iAPF values with a 4 (Task) × 8 (Electrode) × 3 (Group) RM-ANOVA and found faster iAPF during VMIQ-2 relative to BEC (*p*_*Bonf*_ = 0.027), BECM (*p*_*Bonf*_ = 0.003), and KMI (*p*_*Bonf*_ = 0.000) [Task: *F*(3, 168) = 8.912, *p* = 0.000, η^2^ = 0.14]. No other main or interaction effects were significant (Fig. 1). Between-group evaluations of iAPF for each task will be reported in the following sections.

### AO task

**Fig. 2:**
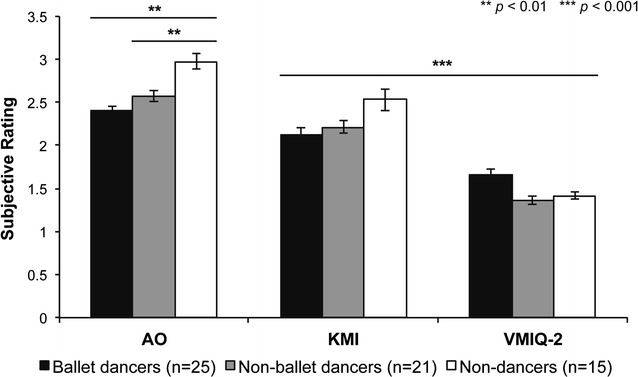
Subjective ratings of action observation and motor imagery. Participants were asked to provide a confidence rating for how accurately they could perform the dance if they were required to for the AO task, and to rate how clearly and vividly they imagined the instructed behaviour for the KMI and VMIQ-2 tasks on a scale from 1 (*Perfectly accurate or clear*) to 5 (*Not at all, or no image at all*). Non-dancers provided significantly higher ratings (i.e., poorer perceived ability) during the AO task relative to ballet dancers (*p* = 0.004) and non-ballet dancers (*p* = 0.044). All groups provided significantly lower ratings, indicative of clearer imagery, when imagining non-expressive movements during the VMIQ-2 task relative to KMI of the newly-learned dance (ballet: *p* = 0.005, non-ballet and non-dance: *p* = 0.000). *Error bars* represent SEM

**Fig. 3:**
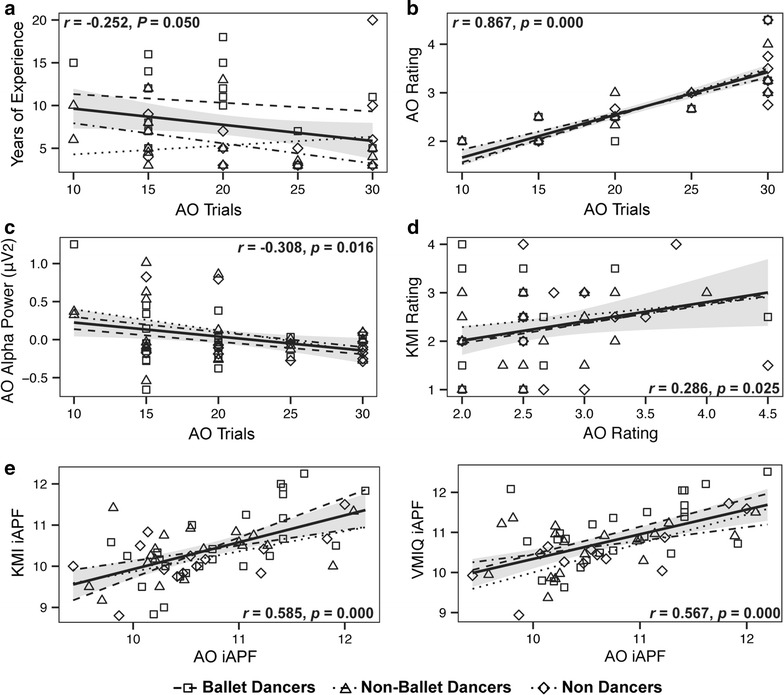
Correlations between behavioral and neural measures of action observation and motor imagery. **a** The number of trials performed during the AO task was negatively correlated to the years of experience participants had in their respective crafts (*p* = 0.050). **b** The number of trials performed during the AO task was positively correlated to the ratings provided at the end of each AO block (*p* = 0.000). **c** During the AO task the number of trials performed was negatively correlated to alpha power (*p* = 0.016). **d** The ratings provided at the end of each AO block were positively correlated to clarity ratings during the KMI task (*p* = 0.025). **e** iAPF was positively correlated between the AO and KMI tasks (*p* = 0.000), and between the AO and VMIQ-2 tasks (*p* = 0.000). Statistical analyses (Pearson correlations) were performed on the average values of each plotted variable collapsed across time (if applicable), electrodes, and groups. Regression lines are shown for visualization purposes only, as the data were not subject to a regression analysis, and include a *solid line* (shaded with a 95% confidence interval) for the overall correlation (i.e., collapsed across all conditions), *dashed lines* for ballet dancers, *dot-dash lines* for non-ballet dancer, and *dotted lines* for non-dancers. Individual data points are represented as *squares* for ballet dancers, *triangles* for non-ballet dancers, and *diamonds* for non-dancers

Empirical behavioural measures of perceived confidence in performing the dance sequence included the number of blocks performed by each subject before moving on to the KMI task, and the average of subjective ratings provided after each block of the AO task. The non-dance group performed significantly more trials (*M* = 24.7, *SD* = 5.8) than both familiar ballet (*M* = 18.8, *SD* = 4.9, *p*_*Bonf*_ = 0.008) and unfamiliar non-ballet dancer groups (*M* = 20.0, *SD* = 6.5, *p*_*Bonf*_ = 0.056) [*F*(2, 58) = 5.170, *p* = 0.009], and were also less confident in their ability to actually perform the dance if required (i.e., higher confidence rating, *M* = 3.0, *SD* = 0.09) compared to ballet (*M* = 2.4, *SD* = 0.05, *p* = 0.004) and non-ballet dance groups (*M* = 2.6, *SD* = 0.06, *p* = 0.004) [*F*(2, 58) = 4.574, *p* = 0.014] (Fig. 2). The number of trials performed was significantly correlated to years of dance experience (*r* = −0.252, *p* = 0.050) (Fig. 3a), subjective ratings (*r* = 0.867, *p* = 0.000) (Fig. 3b), and alpha power (*r* = −0.308, *p* = 0.016) (Fig. 3c). Confidence ratings during the AO task were also significantly correlated to those obtained during the KMI task [*r* = 0.286, *p* = 0.025] (Fig. 3d).

Ballet dancers exhibited faster iAPF (*M* = 10.8, *SD* = 0.7) than both non-ballet dancers (*M* = 10.5, *SD* = 0.7, *p*_*Bonf*_ = 0.023) and non-dancers (*M* = 10.7, *SD* = 0.8, *p*_*Bonf*_ = 0.048) [Group: *F*(2, 25.3) = 5.467, *p*_*HF*_ = 0.011] (Fig. 1), and was higher among ballet dancers than non-ballet dancers during Block 2 (*p*_*Bonf*_ = 0.004) [Group × Time: *F*(8, 18.1) = 3.098, *p*_*HF*_ = 0.022]. A significant main effect of Time [*F*(4, 18.8) = 3.655, *p*_*HF*_ = 0.023] also showed faster iAPF during Block 1 relative to Block 4 (*p*_*Bonf*_ = 0.006), and AO iAPF was significantly correlated to iAPF during KMI [*r* = 0.585, *p* = 0.000] and VMIQ-2 [*r* = 0.567, *p* = 0.000] (Fig. 3e).

**Fig. 4:**
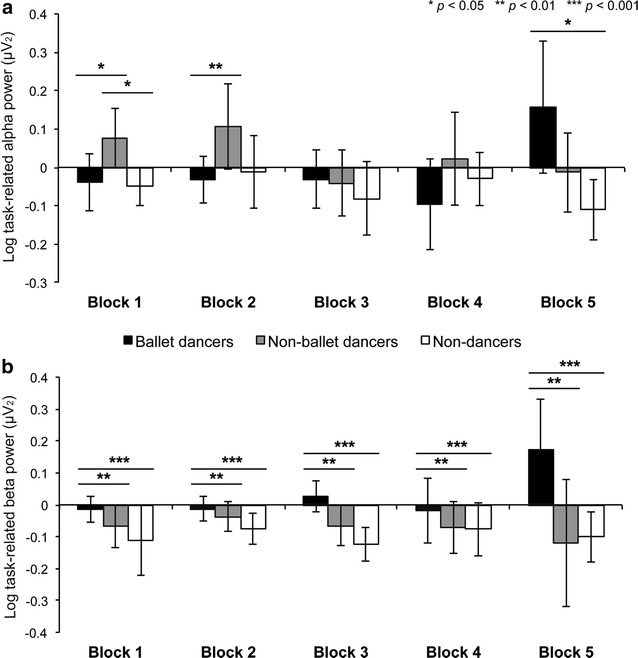
Alpha and beta power during observation of a novel ballet dance sequence. **a** Log task-related (i.e., baseline corrected) alpha power was significantly higher in the non-ballet group relative to non-dancers (*p*_*Bonf*_ = 0.000) in all blocks, while a significant Group × Time interaction revealed higher alpha power in non-ballet dancers than the other groups at Block 1 (ballet: *p*_*Bonf*_ = 0.041, non-dance: *p*_*Bonf*_ = 0.040) and higher than ballet dancers at Block 2 (*p*_*Bonf*_ = 0.010), and greater alpha power in ballet dancers than non-dancers at Block 5 (*p*_*Bonf*_ = 0.039). **b** Log task-related beta power was significantly higher in the ballet dancer group relative to both non-ballet dancers (*p*_*Bonf*_ = 0.003) and non-dancers (*p*_*Bonf*_ = 0.000). *Error bars* represent SEM

Alpha power was greater among non-ballet dancers relative to non-dancers (*p*_*Bonf*_ = 0.000) [Group: *F*(2, 78.8) = 7.704, *p*_*HF*_ = 0.001], but a significant Group × Time interaction [*F*(8, 37.2) = 2.659, *p*_*HF*_ = 0.021] revealed greater alpha power among non-ballet dancers relative to both groups during Block 1 (ballet dancers: *p*_*Bonf*_ = 0.041, non-dancers: *p*_*Bonf*_ = 0.040) and relative to ballet dancers during Block 2 (*p*_*Bonf*_ = 0.010), but greater alpha power among ballet dancers than non-dancers during Block 5 (*p*_*Bonf*_ = 0.039) (Fig. 4a). Alpha and beta power were also significantly correlated during AO (*r* = 0.504, *p* = 0.000). Beta power was significantly greater among ballet dancers relative to both non-ballet dancers (*p*_*Bonf*_ = 0.003) and non-dancers (*p*_*Bonf*_ = 0.000) [Group: *F*(2, 19.0) = 19.810, *p*_*HF*_ = 0.000], demonstrating modulation of beta power by familiarity with the observed stimulus (Fig. 4b). Further, a significant main effect of Electrode [*F*(7, 9.3) = 4.790, *p*_*HF*_ = 0.016] showed higher beta power in bilateral occipital electrodes relative to left premotor cortex (O1: *p*_*Bonf*_ = 0.012, O2: *p*_*Bonf*_ = 0.024).

### KMI task

**Fig. 5:**
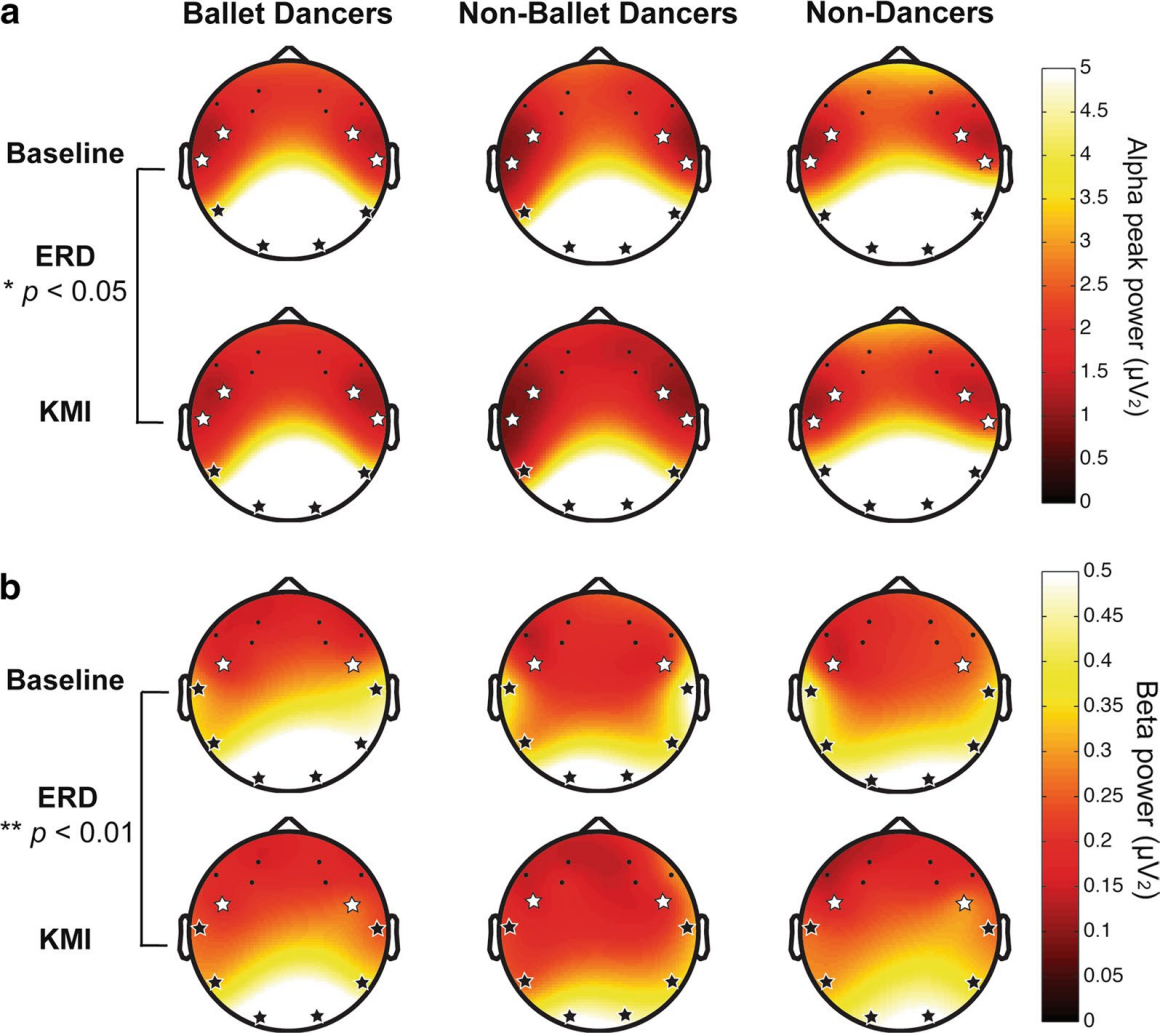
**a** Alpha power during baseline and KMI. Analyses of log alpha peak power during the KMI task and matched baseline condition (eyes closed with music) revealed significant task-related desynchronization (*bottom row*), and a significant main effect of electrode revealed higher alpha power in bilateral occipital and parietal sites (*black stars*) relative to frontocentral and temporal sites (*white stars*). No significant group differences were observed. **b** Beta power during baseline and KMI. Analyses of log beta power during the KMI task and matched baseline condition (eyes closed with music) revealed significant task-related desynchronization (*bottom row*), and a significant main effect of electrode revealed higher alpha power in all posterior sites (*black stars*) relative to frontocentral sites (*white stars*). No significant group differences were observed

Analyses of the average motor imagery ratings provided at the end of each block of the KMI task revealed no differences in clarity or vividness between groups [*F*(2, 58) = 1.137, *p* = 0.328] (Fig. 2), and were significantly correlated to those obtained during the VMIQ-2 task (*r* = 0.370, *p* = 0.003). There were no significant group differences in task-related iAPF (Fig. 1) or alpha power (*p* > 0.05), but comparing log alpha power during KMI to baseline revealed significant desynchronization (baseline > KMI) during motor imagery [Task: *F*(1, 58) = 6.236, *p* = 0.015, η^2^ = 0.098], as well as higher alpha power in O1, O2, P7 and P8 electrodes relative to the temporal and frontal sites [Electrode: *F*(4.7, 272.7) = 207.713, *p*_*HF*_ = 0.000, η^2^ = 0.782] (Fig. 5a). Task-related beta power was higher in left (*p*_*Bonf*_ = 0.001) and right (*p*_*Bonf*_ = 0.013) premotor cortex relative to left superior temporal cortex [Electrode: *F*(4.9, 282.1) = 4.686, *p*_*HF*_ = .000, η^2^ = 0.075]. Similar to alpha power, when comparing log beta power during KMI and baseline we found significant desynchronization during motor imagery [Task: F(1, 58) = 7.951, *p* = 0.007, η^2^ = 0.121] especially in T7 (*p*_*Bonf*_ = 0.000), P7 (*p*_*Bonf*_ = 0.008), O1 (*p*_*Bonf*_ = 0.026), P8 (*p*_*Bonf*_ = 0.006), and T8 (*p*_*Bonf*_ = 0.018) [Task × Electrode: *F*(4.9, 282.1) = 4.686, *p*_*HF*_ = 0.000, η^2^ = 0.075] (Fig. 5b). Beta power was also found to be lower in bilateral premotor cortex relative to all other electrode sites [Electrode: *F*(5.1, 293.7) = 51.496, *p*_*HF*_ = 0.000, η^2^ = 0.470].

### VMIQ-2 task

We confirmed that there were no inherent differences in motor imagery ability between groups with a one-way ANOVA on the average VMIQ-2 scores (i.e., across all 12 movements) [*F*(2, 58) = 2.245, *p* = 0.115] (Fig. 2). Separate 8 (Electrode) × 3 (Group) RM-ANOVAs on iAPF, alpha and beta power, respectively, showed that iAPF was significantly faster among ballet dancers than non-ballet (*p* = 0.043) and non-dancers (*p* = 0.028) [*F*(2,56) = 3.362, *p* = 0.042, η^2^ = 0.107] (Fig. 1), and faster in occipital cortex relative to all other sites [*F*(6.3, 350.3) = 5.843, *p*_*HF*_ = 0.000, η^2^ = 0.094]. Alpha power did not differ between electrodes or groups (*p* > 0.1), and similar to the KMI task beta power was higher in bilateral premotor cortex relative to superior temporal (FC5 > T7: *p* = 0.002, T8: *p* = 0.004; FC6 > T7: *p* = 0.013, T8: *p* = 0.003), left occipital (FC5 > O1: *p* = 0.014; FC6 > O1: *p* = 0.047) and right parietal cortex (FC5 > P8: *p* = 0.005) but only at uncorrected significance levels [Electrode: *F*(5.1, 286.4) = 3.489, *p*_*HF*_ = 0.004, η^2^ = 0.059].

**Fig. 6:**
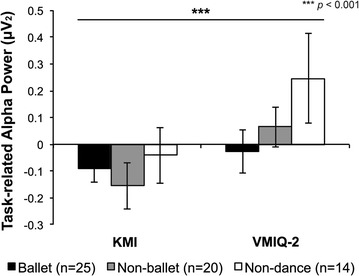
Task-related alpha power during KMI of a newly-learned ballet dance and non-dance movements. Log task-related (i.e., baseline-corrected) alpha power was significantly higher during the VMIQ-2 task than during KMI (*p*_*Bonf*_ = 0. 0 01). Participants performed motor imagery with their eyes closed in both tasks, but showed higher alpha power when imagining non-expressive quotidian movements relative to a newly-learned complex ballet dance sequence. *Error bars* represent SEM

To compare the perceptual quality of KMI for habitual movements to KMI of a complex ballet sequence, we also performed a 2 (Task) x 3 (Group) RM-ANOVA on confidence ratings. It was confirmed that all participants reported significantly clearer and more vivid imagery during the VMIQ-2 task relative to the KMI task^3^ [*F*(1, 58) = 61.821, *p* = 0.000, η^2^ = 0.516, Task × Group: *F*(2, 58) = 3.504, *p* = 0.037, η^2^ = 0.52, ballet dancers: *p*_*Bonf*_ = 0.0005, non-ballet dancers and non-dancers: *p*_*Bonf*_ = 0.000] (Fig. 2). Neural features of KMI (alpha power and beta power) were also compared between the KMI and VMIQ-2 tasks with separate 2 (Task) × 8 (Electrode) × 3 (Group) RM-ANOVAs, respectively. Significantly faster iAPF [*F*(1, 56) = 31.960, *p* = 0.000, η^2^ = 0.363] (Fig. 1) and higher alpha power [*F*(1, 56) = 13.101, *p* = 0.001, η^2^ = 0.19] (Fig. 6) were shown among all participants during the VMIQ-2 task relative to the KMI task. A significant main effect of electrode [*F*(4.8, 266.9) = 2.984, *p*_*HF*_ = 0.005, η^2^ = 0.051] revealed faster iAPF in occipital cortex relative to left premotor cortex (O1: *p*_*Bonf*_ = 0.004, O2: *p*_*Bonf*_ = 0.006), and beta power was higher in premotor cortices than temporal (FC5 > T7: *p*_*Bonf*_ = 0.001, T8: *p*_*Bonf*_ = 0.025; FC6 > T7: *p*_*Bonf*_ = 0.028, T8: *p*_*Bonf*_ = 0.014) and right parietal cortex (FC5 > P8: *p*_*Bonf*_ = 0.006) [Electrode: *F*(4.5, 249.9 = 5.537, *p*_*HF*_ = 0.000, η^2^ = 0.090].

## Discussion

Based on behavioral and neural indices, we demonstrate modulation of oscillatory brain activity by familiarity with an observed ballet stimulus superseding any general dance expertise effects, but no such differences were observed when subjects performed motor imagery of a ballet dance. However, ballet dancers demonstrated faster iAPF during KMI of non-dance movements. Among all participants, KMI was reportedly clearer and more vivid when imagining non-dance movements relative to a complex dance sequence and also elicited faster iAPF and less desychnronization in both alpha and beta frequency bands. These results expand current understanding of experience-dependent plasticity of alpha and beta activity underlying movement processing in dancers. While our conclusions are not based on oscillatory activity in primary motor and/or supplementary motor areas, they provide novel insights on a network of sensorimotor association areas that are modified by learning [28] and expertise [34], and are involved in a variety of functions related to action processing.

Several behavioral indices obtained during the AO task reveal greater task engagement by familiar ballet dancers relative to the unfamiliar non-ballet dancers, who also putatively possess specialized sensorimotor networks arising from dance-related experience but in different genres, and relative to non-dancers, who served as a control group for dance-related expertise and familiarity. Ballet dancers performed significantly fewer blocks of the self-paced task, provided lower subjective ratings reflecting greater confidence in their ability to perform the dance sequence if required (Fig. 2), and a strong positive correlation between these two variables (Fig. 3b). The number of trials performed was also negatively correlated to alpha power (Fig. 3c), demonstrating that subjects who were less confident in their perceived ability to perform the observed task exhibited greater alpha desynchronization than participants who performed fewer trials. Although the clarity ratings provided during the KMI task did not differ between groups, they were positively correlated to AO confidence ratings (Fig. 3d). Examination of the oscillatory activity underlying the experimental tasks will provide greater insight into the mechanisms underlying these behavioural differences in task engagement.

### State iAPF

Higher iAPF is reflected during states of arousal, attention and readiness to perform cognitive tasks including working memory ([17], for review see [14, 35]). However, these evaluations often compare performance outcomes to pre-task iAPF levels obtained at rest. Samaha and Postle [36] compared performance on a two-flash fusion task to pre-task iAPF as well pre-stimulus iAPF, and found that both were correlated to lower thresholds and improved performance on this task, which serves as a measure of temporal resolution of visual perception. In order to form a clear motor image of the dance for the KMI task in the current study, ballet dancers’ visual and sensorimotor systems were able to process more information during AO on a finer temporal scale. Since their iAPF did not differ from other groups in pre-task baseline conditions (Fig. 1), we can infer that this ‘state’ change is driven by a combination of bottom-up perceptual features of the familiar dance genre and top-down influences from existing (embodied) knowledge of the motor repertoire from neural networks shaped by experience [37]. Evidence for the latter is supported by the recent identification of alpha and beta as feedback signals from higher-order regions, including those where the motor representations of ballet movements and positions are coded, to primary sensory areas including the visual cortex [38]. Additionally, it has been noted in dance-related neuroaesthetic literature that dancers, and ballet dancers in particular, use KMI as a tool for mental rehearsal in the absence of overt movement [4, 39]. Although this was not directly assessed in our participants, and no differences in imagery ability were found when comparing VMIQ-2 ratings (Fig. 2), we suggest long-term experience with dance KMI may reinforce the neural pathways that optimally integrate real auditory (i.e., music) information with imagined motor signals [10, 31]. This dance-induced neuroplasticity of oscillatory brain activity may account for the observed increase in iAPF among ballet dancers during VMIQ-2 and AO relative to other groups (Fig. 1). Taken together, results from the AO and VMIQ-2 tasks support and extend previous findings by showing that iAPF can be modulated by experience in dance, and specifically in ballet.

### Engagement versus difficulty: iAPF and alpha power

Haegens et al. [18] provide evidence for modulation of iAPF *during* task performance, with increased cognitive demand resulting in faster occipital and parietal iAPF. Accordingly, one would expect higher iAPF while visualizing a more demanding action (dance) relative to non-dance movements (VMIQ-2). However, the opposite pattern was observed suggesting all subjects were more engaged during KMI of the less demanding VMIQ-2 task (Fig. 1). Conflicting evidence for changes in alpha *power* that accompany higher iAPF have been shown depending on the nature of the cognitive task; desynchronization has been shown to reflect the active engagement in encoding and memory retention [40] while alpha synchronization (increased alpha power) suggests a decrease in expended cortical resources following skill learning [41] and is related to suppression of distracting information that can interfere with encoding from parieto-occipital sources [42, 43] that comprise most of the source signals in the current investigation. As such, the desynchronization elicited during dance KMI may reflect processing of the dance that had just been observed and encoded in working (visual) memory while the relatively greater alpha power elicited during the VMIQ-2 task could reflect the reduced cognitive load for imagining simple movements (Table 1) in the face of competing stimuli or attentional demands (Fig. 6). This pattern is consistent with the neural efficiency hypothesis [44], which supposes that alpha synchronization reflects inhibition of task-irrelevant areas, including posterior visual areas when performing first-person KMI [24, 45], and alpha desynchronization is highest during focused task-related activation such as what would be expected during KMI of a newly-learned task. Thus, engaging with familiar stimuli recruits oscillatory mechanisms that are distinct from those that are phenomenologically similar to processes engaged during very difficult tasks.

### Neural efficiency: alpha and beta power

Further evidence for the neural efficiency hypothesis can be observed during the AO task, which replicates previous evidence for experience-dependent alpha desynchronization during AO [22] but with evidence for desynchronization among non-dancers as well (Fig. 4a). To confirm that this similar pattern of alpha power among groups on opposite ends of the familiarity continuum does not reflect the same underlying process, we can consider the observed beta power results during AO. Beta desynchronization has been shown to accompany alpha desynchronization during AO (lower beta (13–18 Hz) only in [22]) and KMI [46], and is further decreased during KMI and execution of skilled movements [47, 48]. In addition, both frequency and power of the beta band have been related to cognitive aspects of movement, including cue anticipation or expectation, visuomotor integration and preparation [49]. Beta synchronization is typically observed during the ‘post-movement rebound’ in a wider distributed premotor and sensorimotor area [50–52] and occurs immediately following real or imagined movement as a ‘resetting’ of motor representations in these networks [53–55]. If beta oscillations originate in the primary motor cortex as previously suggested [48, 50], and responsivity of the sensorimotor cortex is greater for familiar embodied actions [6–9, 56], then perhaps the degree of beta desynchronization can also be nuanced by experience-dependent plasticity. Because our current sample of ballet dancers exhibit significantly greater familiarity and expertise (i.e., more years of experience) in dance, the influence of these factors taken together may compound the observed beta desynchronization during AO relative to both unfamiliar groups (Fig. 4b). From a neurophysiological perspective, these findings could possibly reflect less suppression of the distributed sensorimotor network that encodes existing genre-specific motor representations, which would only provide competition for limited neural resources among less experienced and unfamiliar dancers. Thus, by considering activity in multiple frequency bands during the same task, the present results clarify the impact of familiarity and expertise more generally on multiple concurrent functional roles for alpha and beta power during AO and KMI.

### Therapeutic applications for dance-induced modulation of sensorimotor oscillations

A growing body of evidence is lending credit to the therapeutic benefits of dance practice on general health, and especially for the elderly and those afflicted with movement disorders (for review, see [57]). It is well established that iAPF is lower in the elderly [16] and especially among those with neurological disorders like Alzheimer’s [58] and Parkinson’s disease (PD) [59, 60]. Ongoing investigations by our lab and other research teams are demonstrating efficacy of Dance with Parkinson’s programs on the neural, motor and non-motor symptoms of PD, including increased global alpha power following a single dance class [26, 61, 62]. The presently demonstrated state/task-related increases in iAPF during KMI of everyday movements provides encouraging evidence for low-impact KMI training programmes that can yield improvements in iAPF functioning that can translate to resting state iAPF improvements. Previous work from our lab reveals dance-induced plasticity in the extrastriate body area during movement among expert dancers [11], which has been shown to serve as an alternate motor pathway for people with PD [63, 64]. Further research on how dance-induced neuroplasticity manifests in healthy experts will provide greater insights to effective interventions for this and other clinical populations.

## Conclusions

Several important insights are revealed in light of the observed outcomes, including a further understanding of experience-dependent plasticity of the neural mechanisms underlying action observation and KMI of specialized and habitual movements. Specifically, we demonstrate modulation of alpha peak frequency and power in the alpha and beta frequency bands during observation of a familiar dance sequence relative to unfamiliar participants, with further expertise effects resulting in more efficient visuomotor processing during KMI of non-dance movements. While all participants demonstrated alpha and beta desynchronization during KMI, we clarify that familiarity with the imagined stimulus does not result in significant modulation of these signals, unlike action observation. Together, these findings promote the translational benefits of dance practice to attentional and sensorimotor networks, and lend further support for the efficacy of dance and motor imagery as effective BCI source signals and therapeutic interventions for clinical populations with movement disorders.

## Abbreviations

fMRI: functional magnetic resonance imaging
EEG: electroencephalography
KMI: kinaesthetic motor imagery
AO: action observation
VMIQ-2: Vividness of Movement Imagery Questionnaire 2

## Footnotes

1 Although the ballet group has significantly more years of experience than non-ballet (*p*_Bonf_ = 0.000) and non-dance groups (*p*_Bonf_ = 0.002), no dependent variables were significantly correlated with years of experience except for the number of trials performed during the AO task (see “Results: AO task” section).

2 Two subjects from the non-dance group did not have iAPF values for the VMIQ-2 task, but had iAPF values and were included in analyses for the AO and KMI tasks.
